# Supplementary material for: Deciphering early human pancreas development at the single-cell level
Source: Nat Commun. 2023 Sep 2;14:5354. doi: 10.1038/s41467-023-40893-8 (PMC10475098; doi:10.1038/s41467-023-40893-8)
Supplement: Supplementary file 1 — Supplementary Information [file 41467_2023_40893_MOESM1_ESM.pdf]

Supplemental information

Deciphering early human pancreas development at the single-cell level

Zhuo Ma[1,2#], Xiaofei Zhang[1,3,4#], Wen Zhong[5,6#], Hongyan Yi[4], Xiaowei Chen[7], Yinsuo Zhao[1], Yanlin Ma[4*], Eli Song[1*], Tao Xu[1,2,8,9,10*]

[1]National Laboratory of Biomacromolecules, CAS Center for Excellence in Biomacromolecules, Institute of Biophysics, Chinese Academy of Sciences, Beijing 100101, China

[2]College of Life Sciences, University of Chinese Academy of Sciences, Beijing 100049, China

[3]Key Laboratory of Molecular Biophysics of the Ministry of Education, College of Life Science and Technology, Huazhong University of Science and Technology, Wuhan 430074, China

[4]Hainan Provincial Key Laboratory for Human Reproductive Medicine and Genetic Research, Key Laboratory of Reproductive Health Diseases Research and Translation (Hainan Medical University), Ministry of Education, The First Affiliated Hospital of Hainan Medical University, Hainan Medical University, Haikou 570102, China

[5]Science for Life Laboratory, Department of Biomedical and Clinical Sciences (BKV), Linköping University, Linköping 581 83, Sweden

[6]Department of Neuroscience, Karolinska Institutet, Stockholm, Sweden

[7]Center for High Throughput Sequencing, Core Facility for Protein Research, Key Laboratory of RNA Biology, Institute of Biophysics, Chinese Academy of Sciences, Beijing 100101, China

[8]Guangzhou Laboratory, Guangzhou, China, Guangzhou 510005, China

[9]Central Hospital Affiliated to Shandong First Medical University, Jinan 250013, China.

[10]Medical Science and Technology Innovation Center, Shandong First Medical University & Shandong Academy of Medical Sciences, Jinan 250062, China.

*Correspondence: xutao@ibp.ac.cn; songali@ibp.ac.cn; mayanlinma@hotmail.com

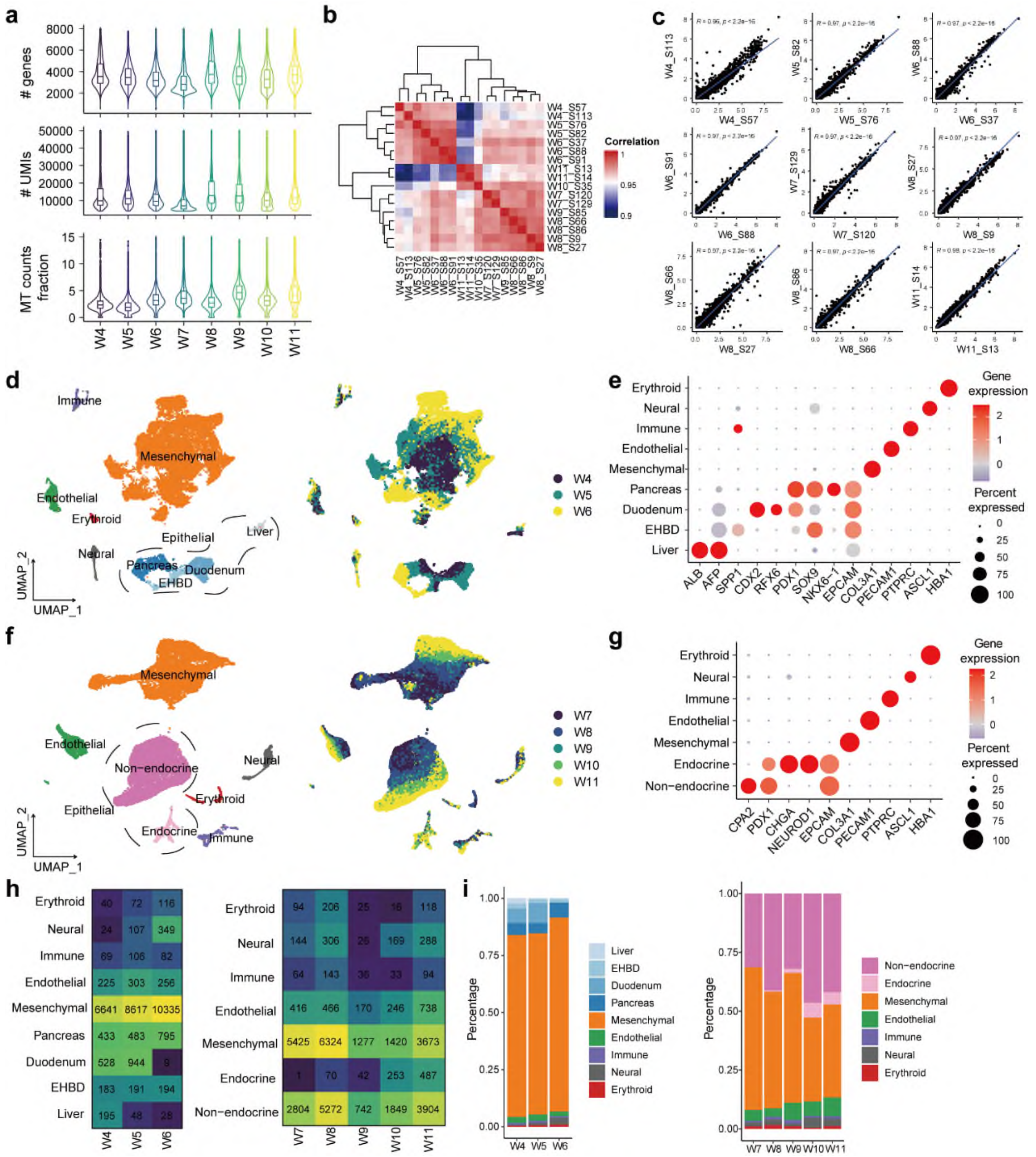

Supplementary Fig. 1. Data quality of scRNA-seq data and marker gene expression, related to Fig. 1.

a Violin plot combined with box plot showing the distribution of the number of genes, the number of unique member identifiers (UMIs), and the mitochondrial (MT) gene fraction per cell grouped by PCW. The center line, bounds of box, whiskers, and single points represent median, 25th to 75th percentile range, 5th and 95th percentile range as well as outliers. W4, n = 8338 cells; W5, n = 10871 cells; W6, n = 12164 cells; W7, n = 8948 cells; W8, n = 12787 cells; W9, n = 2318 cells; W10, n = 3986 cells; W11, n = 9302 cells. **b** Heatmap showing Pearson's correlation coefficient between the transcriptomes of each sample. Pearson's correlation coefficient was calculated by average expression value of each gene in each sample. **c** Scatter plot of gene expression between different samples at the same time point. **d** UMAP plot of all single-cells colored by cell class and time point in the PCW 4-6 samples. EHBD, extrahepatic bile ducts. **e** Dot plot showing cell-class-specific marker

gene expression in PCW 4-6 samples. **f** UMAP plot of all single-cells colored by cell class and time point in the PCW 7-11 samples. **g** Dot plot showing cell-class-specific marker gene expression in PCW 7-11 samples. **h** Heatmap showing the cell numbers of each cell class cluster in the PCW 4-6 and PCW 7-11 samples. **i** Bar plot showing the percentage of each cell class in the PCW 4-6 and PCW 7-11 samples.

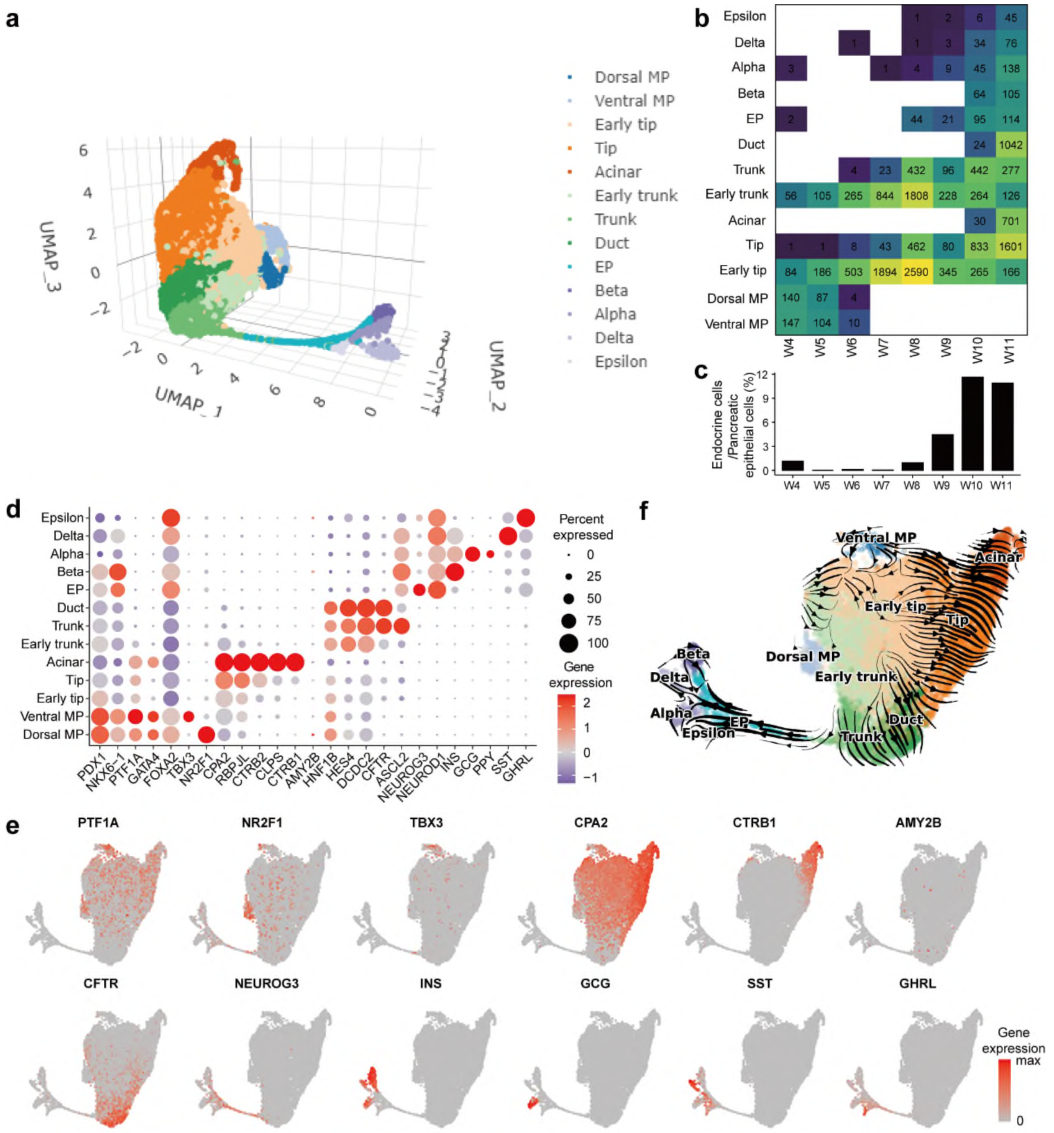

Supplementary Fig. 2. Molecular diversity of early human pancreatic epithelial cells, related to Fig. 1.

a 3D UMAP plot of all single cells colored by cell type in pancreatic epithelial cells. **b** Heatmap showing the cell numbers of each cell type in pancreatic epithelial cells. **c** Bar plot showing the percentage of endocrine cells in the whole pancreatic epithelial cells. **d** Dot plot showing cell type marker gene expression in pancreatic epithelial cells. **e** Feature plot showing the expression of other key marker genes of pancreatic epithelial cells. **f** RNA velocity plot showing the developmental trajectory of pancreatic epithelial cells.

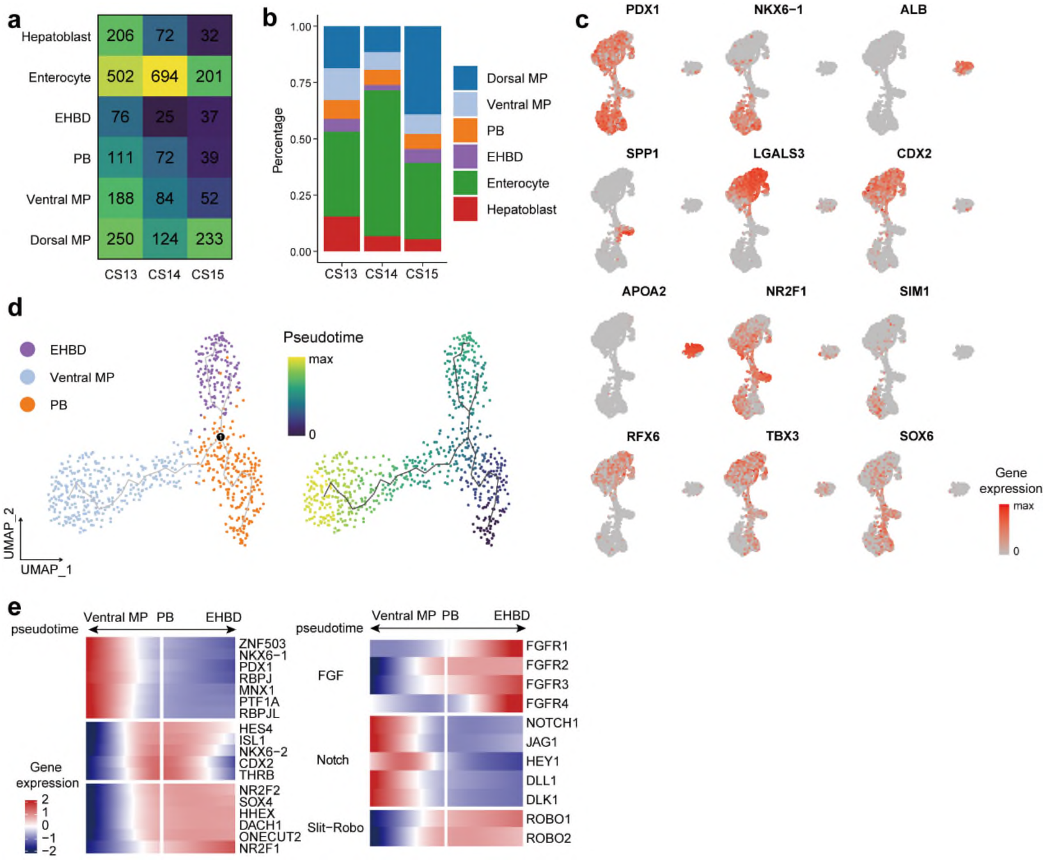

Supplementary Fig. 3. Transcriptional heterogeneity of early human pancreas adjacent epithelial cells, related to Fig. 2.

a Heatmap showing the cell numbers of each cell type in the early epithelial cells. **b** Bar plot showing the percentage of each cell type in the early epithelial cells. **c** Feature plot showing the expression of key marker genes in Fig. 2e. **d** UMAP plot showing the developmental trajectory of PB, EHBD, and ventral MP cells. **e** Heatmap showing the bifurcation of gene expression along the developmental trajectory of PB progenitors committed to EHBD and ventral MP fates.

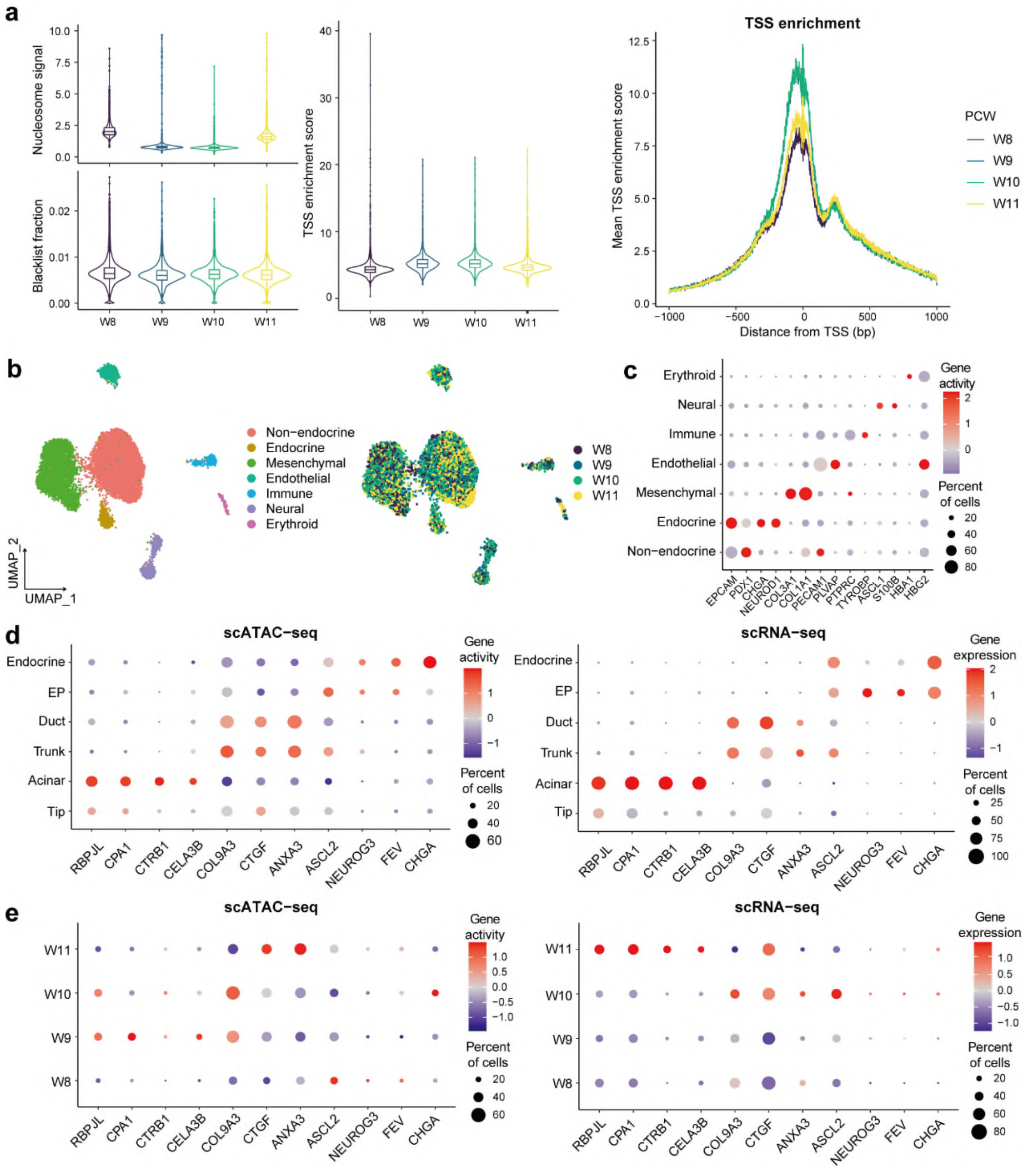

Supplementary Fig. 4. Quality control of scATAC-seq data, related to Fig. 3.

a Violin plot combined box plot showing the nucleosome signal, blacklist ratio and TSS enrichment per cell grouped by PCW. TSS, transcription start site. The center line, bounds of box, whiskers, and single points represent median, 25th to 75th percentile range, 5th and 95th percentile range as well as outliers. W8, n = 7004 cells; W9, n = 3848 cells; W10, n = 5465 cells; W11, n = 5786 cells. **b** UMAP plot of all single cells colored by cell class and time point in all cells. **c** Dot plot showing the accessibility and expression of markers for each cell class. **d** Dot plot showing the accessibility and expression of markers for each cell type. **e** Dot plot showing the accessibility and expression of markers for each cell class at different time points.

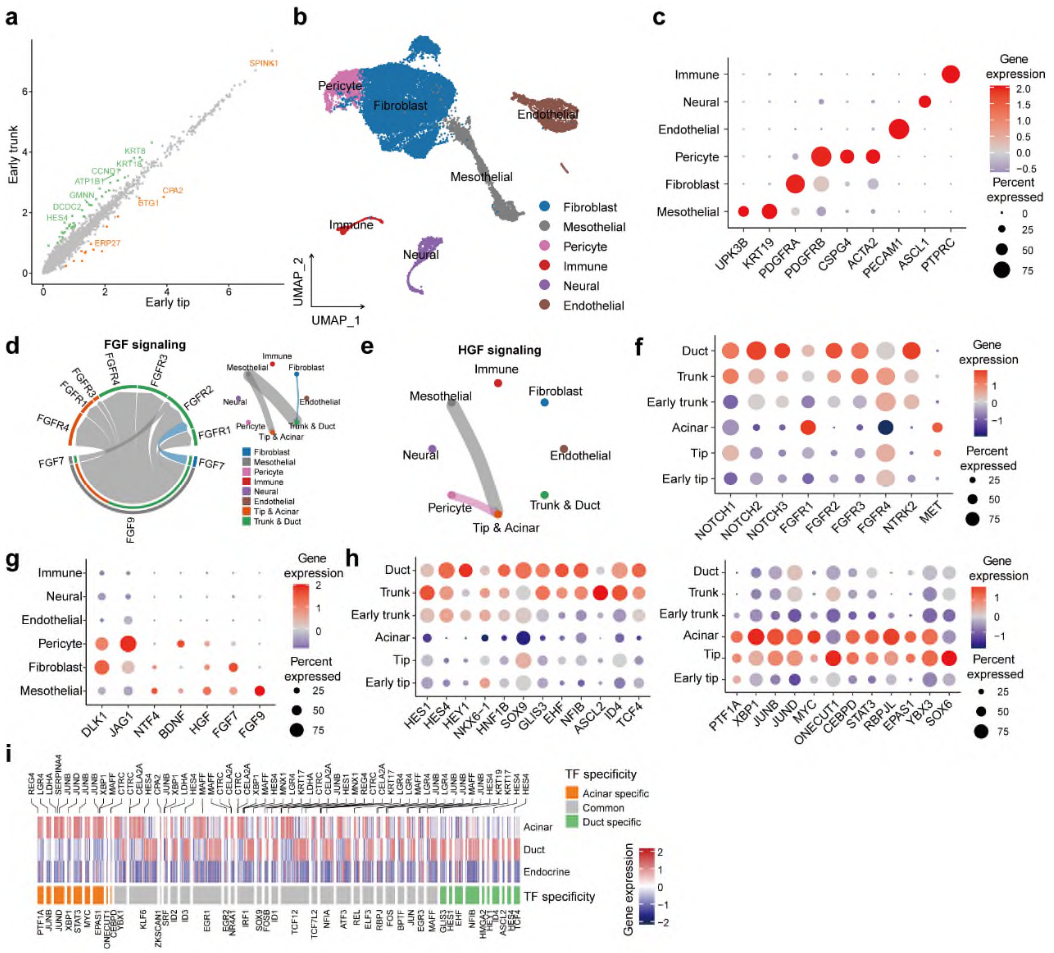

Supplementary Fig. 5. Cell fate divergence of acinar and ductal lineage cells, related to Fig. 4.

a Scatter plot showing the mean expression of differentially expressed genes between early trunk and early tip cells. **b** UMAP plot of supporting cell colored by cell type in the PCW 7-11 samples. **c** Dot plot showing cell-type-specific marker gene expression in supporting cells. **d** Network plot showing FGF signaling pathway-related interactions between acinar, ductal lineage cells and supporting cells. **e** Network plot showing HGF signaling interactions between acinar, ductal lineage cells and supporting cells. **f** Dot plot showing related receptor gene expression in ductal lineage and acinar lineage cells. **g** Dot plot showing related ligand gene expression in supporting cells. **h** Dot plot showing related TF expression in ductal lineage and acinar lineage cells. **i** Heatmap showing the expression of targets of the three sets of TFs.

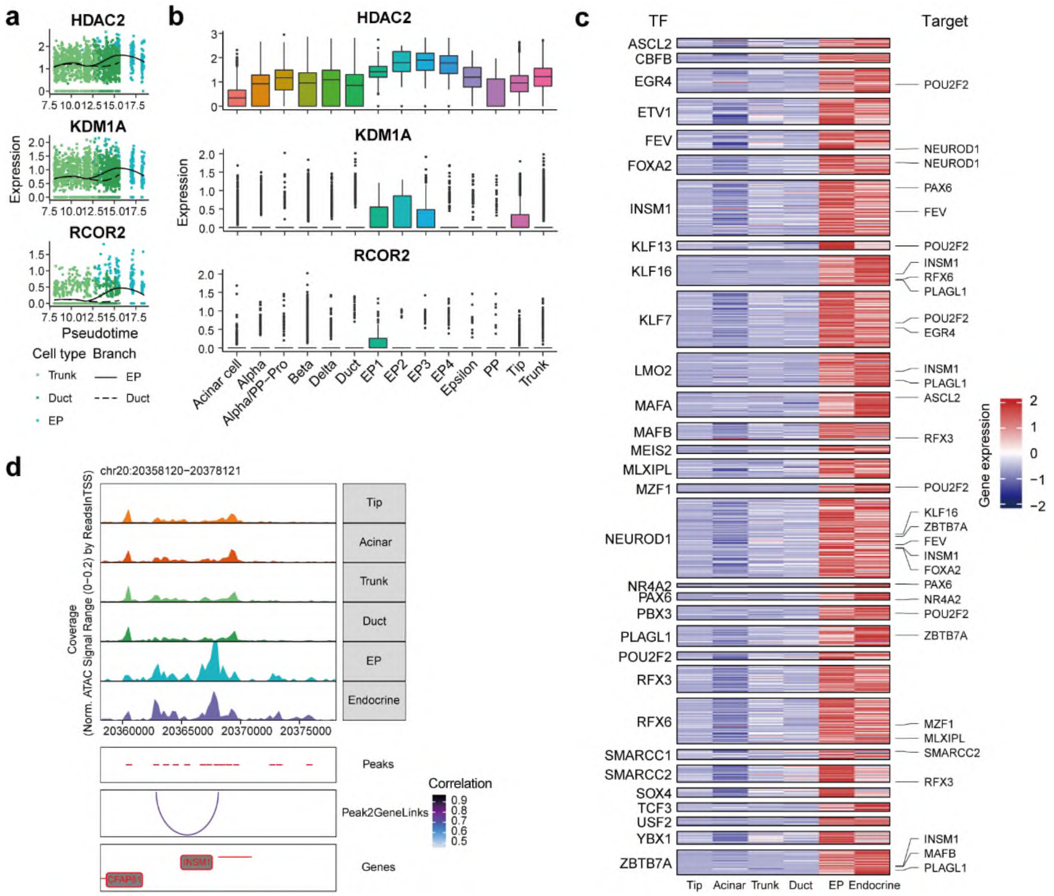

Supplementary Fig. 6. Transcriptional heterogeneity of trunk, duct and endocrine progenitors, related to Fig. 5.

a Gene expression dynamic of some epigenetic regulation enzymes in duct and EP cell differentiation. **b** Box plot showing the expression of some epigenetic regulation enzymes in another dataset. The center line, bounds of box, whiskers, and single points represent median, 25th to 75th percentile range, 5th and 95th percentile range as well as outliers. Acinar cell, n = 544 cells; Alpha, n = 302 cells; Alpha/PP-Pro, n = 75 cells; Beta, n = 843 cells; Delta, n = 211 cells; Duct, n = 241 cells; EP1, n =25 cells; EP2, n = 20 cells; EP3, n = 46 cells; EP4, n = 84 cells; Epsilon, n = 206 cells; PP, n = 17 cells; Tip, n =518 cells; Trunk, n = 585 cells. **c** Expression patterns of targets of endocrine TFs. **d** Peak accessibility in selected regions.

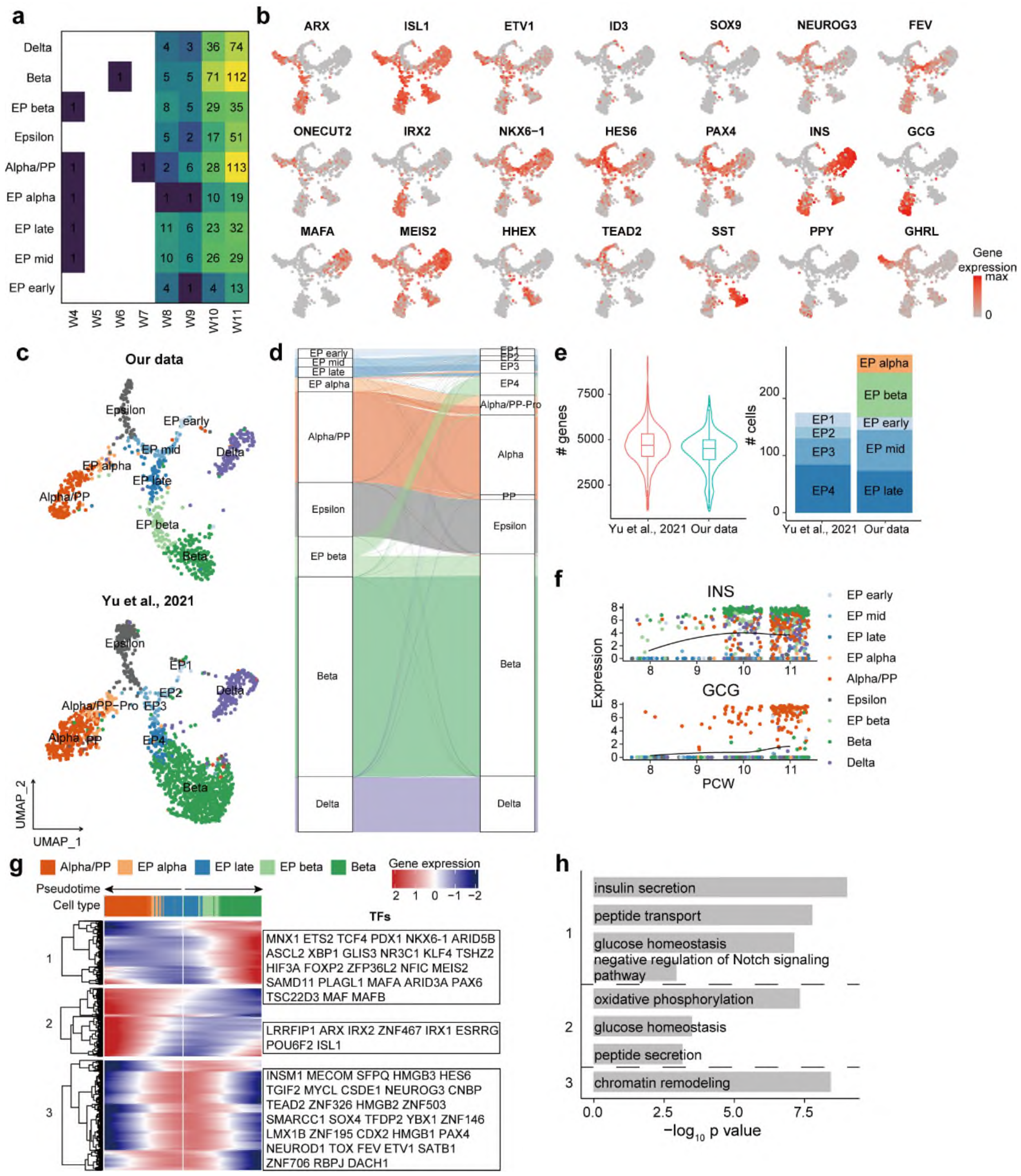

Supplementary Fig. 7. Molecular diversity of subpopulations in human developing pancreatic endocrine cells, related to Fig. 6.

a Heatmap showing the cell numbers of each cell type in developing pancreatic endocrine cells. **b** Feature plot showing marker gene expression in developing pancreatic endocrine cells. **c** UMAP plot showing integration of our dataset and another human developing pancreatic endocrine cell dataset. **d** Alluvial plot illustrating the relationship between our dataset and another human developing pancreatic endocrine cell dataset. **e** Violin plot combined box plot showing the distribution of the number of genes per cell. The center line, bounds of box, whiskers, and single points represent median, 25th to 75th percentile range, 5th and 95th percentile range as well as outliers. Yu's dataset, n = 1829 cells; our dataset, n = 814 cells. Bar plot showing the cell numbers of EP subclusters grouped by different datasets. **f** Gene expression dynamics of insulin and glucagon along the PCW in pancreatic endocrine cells. **g** Gene expression dynamics of marker genes in developing

alpha and beta cells. **h** Selected enriched GO terms of genes in Supplementary Fig. 7g. P values were calculated by using *enrichGO* function from R package clusterProfiler with one-sided hypergeometric test.
