# Supplementary material for: Deciphering early human pancreas development at the single-cell level
Source: Nat Commun. 2023 Sep 2;14:5354. doi: 10.1038/s41467-023-40893-8 (PMC10475098; doi:10.1038/s41467-023-40893-8)
Supplement: Supplementary file 3 — Description of Additional Supplementary Files [file 41467_2023_40893_MOESM3_ESM.pdf]

Description of Additional Supplementary Files

Supplementary Data 1. Human embryo sample information

Supplementary Data 2. Differentially expressed genes of pancreatic epithelial cells, related to Fig. 1.

Supplementary Data 3. Differentially expressed genes of epithelial cells in PCW 4 and 5, related to Fig. 2.

Supplementary Data 4. Differentially accessible peaks of pancreatic epithelial cells, related to Fig. 3.

Supplementary Data 5. Differentially expressed genes of pancreatic endocrine cells, related to Fig. 6.

Supplementary Data 6. Differentially expressed regulons of pancreatic endocrine cells, related to Fig. 6.
