# Supplementary material for: Deciphering early human pancreas development at the single-cell level
Source: Nat Commun. 2023 Sep 2;14:5354. doi: 10.1038/s41467-023-40893-8 (PMC10475098; doi:10.1038/s41467-023-40893-8)
Supplement: Supplementary file 10 — Reporting Summary [file 41467_2023_40893_MOESM10_ESM.pdf]

nature portfolio

Corresponding author(s): Tao Xu

Last updated by author(s): Apr 24, 2023

Reporting Summary

Nature Portfolio wishes to improve the reproducibility of the work that we publish. This form provides structure for consistency and transparency in reporting. For further information on Nature Portfolio policies, see our Editorial Policies and the Editorial Policy Checklist.

Statistics

For all statistical analyses, confirm that the following items are present in the figure legend, table legend, main text, or Methods section.

n/a	Confirmed	
☐	☒	The exact sample size (*n*) for each experimental group/condition, given as a discrete number and unit of measurement
☐	☒	A statement on whether measurements were taken from distinct samples or whether the same sample was measured repeatedly
☐	☒	The statistical test(s) used AND whether they are one- or two-sided<br>*Only common tests should be described solely by name; describe more complex techniques in the Methods section.*
☒	☐	A description of all covariates tested
☐	☒	A description of any assumptions or corrections, such as tests of normality and adjustment for multiple comparisons
☐	☒	A full description of the statistical parameters including central tendency (e.g. means) or other basic estimates (e.g. regression coefficient) AND variation (e.g. standard deviation) or associated estimates of uncertainty (e.g. confidence intervals)
☐	☒	For null hypothesis testing, the test statistic (e.g. *F*, *t*, *r*) with confidence intervals, effect sizes, degrees of freedom and *P* value noted<br>*Give P values as exact values whenever suitable.*
☒	☐	For Bayesian analysis, information on the choice of priors and Markov chain Monte Carlo settings
☒	☐	For hierarchical and complex designs, identification of the appropriate level for tests and full reporting of outcomes
☐	☒	Estimates of effect sizes (e.g. Cohen's *d*, Pearson's *r*), indicating how they were calculated

Our web collection on statistics for biologists contains articles on many of the points above.

Software and code

Policy information about availability of computer code

Data collection	Sequencing library was constructed by Chromium Next GEM Single Cell 3' Reagent Kits v3.1 (10x Genomics) and Chromium Next GEM Single Cell ATAC Reagent Kits v1.1 (10x Genomics) according to the manufacturer's instructions. And then the library was processed on the Illumina NovaSeq6000 platform for sequencing. Imaging data was collected from Olympus FV3000 confocal microscope.
Data analysis	CellRanger(4.0.0), CellRanger ATAC(2.0.0), R(4.1.2), SoupX(1.5.2), Seurat(4.0.5), harmony(0.1.0), Monocle3(1.0.0), velocyto.py(0.17), scVelo (0.2.4), ClusterProfiler(4.2.0), CellChat(1.1.3), ggplot2(3.3.5), ComplexHeatmap(2.13.1), pySCENIC(0.11.2), Signac(1.6.0), ArchR(1.0.1), chromVARmotifs(0.2.0), MACS2(2.1.2), TOBIAS(0.13.3), ImageJ(1.52). See the Methods for details.

For manuscripts utilizing custom algorithms or software that are central to the research but not yet described in published literature, software must be made available to editors and reviewers. We strongly encourage code deposition in a community repository (e.g. GitHub). See the Nature Portfolio guidelines for submitting code & software for further information.

Data

Policy information about availability of data

All manuscripts must include a data availability statement. This statement should provide the following information, where applicable:

- Accession codes, unique identifiers, or web links for publicly available datasets
- A description of any restrictions on data availability
- For clinical datasets or third party data, please ensure that the statement adheres to our policy

The raw sequencing data generated in this study have been deposited in the Genome Sequence Archive for Human (GSA-Human) under accession code HRA002757.

The data in GSA are available under restricted access for privacy protection, access can be obtained by contacting Tao Xu (xutao@ibp.ac.cn). The processed gene expression matrix for scRNA-seq and Tn5 fragment files and filtered peak-barcode matrix for scATAC-seq data are available at OMIX database under accession code OMIX001616. Source data are provided with this paper. Other published datasets we used in this study could be obtained from GSE115931, GSE139627 and OMIX236. The GRCh38 (hg38) reference genome was used from 10x Genomics (https://support.10xgenomics.com/single-cell-gene-expression/software/downloads/latest). The CellChatDB.human database was used from CellChat(1.1.3) package.

Human research participants

Policy information about studies involving human research participants and Sex and Gender in Research.

Reporting on sex and gender	For scRNA-seq, we collected human embryonic pancreas samples at 8 time points from post-conception week (PCW) 4 to 11 from 17 donors, including 6 males and 11 females. For scATAC-seq, we collected human embryonic pancreas samples at 4 time points from PCW 8 to 11 from 4 donors, including 1 males and 3 females. Detailed sex information of human embryo are reported in Supplementary Data 1. And sex was not considered in this study design and analyses based on sex were not performed due to insufficient samples.
Population characteristics	Other information of human embryo are also reported in Supplementary Data 1.
Recruitment	The patients who take voluntary abortions in The First Affiliated Hospital of Hainan Medical University were included after informed consent. The post-conception age of embryos ranged from 4 to 11 weeks.
Ethics oversight	Ethics Committee of The First Affiliated Hospital of Hainan Medical University (#201901)

Note that full information on the approval of the study protocol must also be provided in the manuscript.

Field-specific reporting

Please select the one below that is the best fit for your research. If you are not sure, read the appropriate sections before making your selection.

☒ Life sciences ☐ Behavioural & social sciences ☐ Ecological, evolutionary & environmental sciences

For a reference copy of the document with all sections, see nature.com/documents/nr-reporting-summary-flat.pdf

Life sciences study design

All studies must disclose on these points even when the disclosure is negative.

Sample size	For scRNA-seq, We collected human embryonic pancreas samples at 8 time points from post-conception week (PCW) 4 to 11 from 17 donors. For each sample, we collected 5000-8000 cells for library construct.<br>For scATAC-seq, we collected human embryonic pancreas samples at 4 time points from PCW 8 to 11 from 4 donors. For each sample, we collected 3000-6000 nuclei for library construct.
Data exclusions	We exclude the cells that failed quality control. The criteria are detailed in the Methods.
Replication	For scRNA-seq, Two or three embryonic pancreas were collected from different embryos at the same developmental stage (PCW4,5,6,7,8,11) to exclude the potential batch effect and individual differences. For scATAC-seq, only one embryonic pancreas was collected at the same developmental stage due to the scarcity of samples. Immunofluorescence images represent the results from 3 experiments. All replications were consistent for results.
Randomization	The embryos in this study were from pregnant women who take voluntary abortions. In addition, single cells or single nuclei of collected samples were randomly picked, and the suspension was homogeneous with no artificial preference.
Blinding	Blinding was not relevant to this study since no specific grouping.

Reporting for specific materials, systems and methods

We require information from authors about some types of materials, experimental systems and methods used in many studies. Here, indicate whether each material, system or method listed is relevant to your study. If you are not sure if a list item applies to your research, read the appropriate section before selecting a response.

Materials & experimental systems

n/a	Involved in the study
☐	☒ Antibodies
☒	☐ Eukaryotic cell lines
☒	☐ Palaeontology and archaeology
☒	☐ Animals and other organisms
☒	☐ Clinical data
☒	☐ Dual use research of concern

Methods

n/a	Involved in the study
☒	☐ ChIP-seq
☒	☐ Flow cytometry
☒	☐ MRI-based neuroimaging

Antibodies

Antibodies used

Mouse anti-DCDC2 (C-4) (1:50, Santa Cruz, sc-166051), Mouse anti-ASCL2 (7E2) (1:100, Millipore, MAB4418), Rabbit anti-PDX1 (EPR22002) (1:100, Abcam, ab219207), Rabbit anti-HES4 (1:100, Invitrogen, PA5-84551), Goat anti-Mouse IgG (H+L) Cross-Adsorbed Secondary Antibody, Alexa Fluor 488 (1:400, Invitrogen, A-11001), Donkey anti-Rabbit IgG (H+L) Highly Cross-Adsorbed Secondary Antibody, Alexa Fluor 568 (1 : 400, Invitrogen, A-10042)

Validation

All antibodies used in this study were obtained from commercial source, and validated according to manufacturers' instruction.
